# Human Interleukin 23 Receptor Induces Cell Apoptosis in Mammalian Cells by Intrinsic Mitochondrial Pathway Associated with the Down-Regulation of RAS/Mitogen-Activated Protein Kinase and Signal Transducers and Activators of Transcription factor 3 Signaling Pathways

**DOI:** 10.3390/ijms141224656

**Published:** 2013-12-18

**Authors:** Wei-Ye Shi, Chang-Yan Che, Li Liu

**Affiliations:** Department of Microbiology, Institute of Basic Medical Sciences, Chinese Academy of Medical Sciences & Peking Union Medical College, Beijing 100005, China; E-Mails: feiwudeyezi@126.com (W.-Y.S.); ccyckk@163.com (C.-Y.C.)

**Keywords:** IL-23R, apoptosis, intrinsic pathway, RAS/MAPK, STAT3

## Abstract

The composition of IL-23R complex is similar to that of the IL-12 receptor (IL-12R) complex with a shared IL-12R-β1 chain. The IL-12R-β1 heterodimerizes with IL-23R and IL-12R-β2 to form IL-23R and IL-12R complexes, respectively. The IL-12R-β2 has been shown to function as a tumor suppressor gene and apoptotic inducer. However, whether IL-23R also functions in cell apoptosis is currently unknown. In this study, we demonstrate for the first time that overexpression of IL-23R markedly induces cell apoptosis in both 293ET and HeLa cells. The activations of caspase 3 and caspase 9 are induced by IL-23R. Mechanistic study reveals that IL-23R markedly inhibits RAS/MAPK and STAT3 but not STAT1 and PI-3K/Akt signaling pathways in both 293ET and HeLa cells. Overexpression of IL-23R significantly up-regulates IL-12Rβ1 expression but not IL-23α and IL-12β expressions in both cell lines. Therefore, our data strongly indicates that IL-23R is able to induce cell apoptosis by activating the intrinsic mitochondrial pathways associated with the inhibition in RAS/MAPK and STAT3 activations in mammalian cells.

## 1. Introduction

The interleukin 12 (IL-12) family consists of at least four members of cytokines (IL-12, IL-23, IL-27 and IL-35) that play diverse roles in different subtypes of T lymphocytes, and is the only cytokine family that forms a functional α or β heterodimer [1–3]. The α subunit of IL-12 family includes p 19, p 28 and p 35 chains that are individually paired with the β subunit (p 40 or Ebi3) of IL-12 family. The combinations of the paired α/β chains for IL-12, IL-23, IL-27 and IL-35 are p 35/p 40, p 19/p 40, p 28/Ebi3 and p 35/Ebi3, respectively [2,3]. The above paired ligands can be specifically recognized by a shared array of heterodimerized receptors. Interleukin 12 (IL-12) signals IL-12Rβ1 and IL-12Rβ2 [4,5], whereas IL-23 signals IL-12Rβ1 and IL-23R [6,7]. Differently, IL-27 recognizes WSX-1 (IL-27R) and gp130 [8], whereas IL-35 binds to gp130 and IL-12β2 [9]. Although structurally related, the members of IL-12 family even conduct distinct immune functions. Both IL-12 and IL-23 can be released by the major antigen presenting cells (APCs) such as dendritic cells and macrophages, *etc*., into the inflammatory sites upon microbial infection. A positive feedback loop is required for continuous IL-12 production by APCs as well as for the IFN-γ mediated T_H_1 differentiation. Similarly, a positive feedback loop is also found in the regulation of IL-23 production by enhancing the IL-23R expression. Unlike IL-12, IL-23 does not mediate cell differentiation but can positively regulate IL-17- T_H_17 cell pathway. In contrast, both IL-27 and IL-35 possess inhibitory properties to limit or counteract the cellular inflammatory responses.

The expression of IL-12 receptor family was initially thought to be restricted in immune cells. However, broader examinations on a variety of tissues indicated that the expressions of the members of IL-12/IL-12R family could be regulated in many non-immune tissues and cells including human cancers [10]. Recent study shows that IL-23 regulates the proliferation versus inhibition of IL-23R positive lung cancer cells in a concentration dependent manner [11]. However, in most instances, genetic defects, structural defects and/or the gene expression defects in IL-12/IL12R family members might be the causing issues for the development of a variety of human cancers such as esophageal cancer [12], lung cancer [10], acute myeloid leukemia [13], oesophageal squamous cell carcinoma [14] and human chronic B cell malignancies [15]. Therefore, supplementing a functional IL-12/IL-12R family gene into the disease tissues might generate great therapeutic potential.

Mounting evidence has shown that some IL-12/IL12R family members possess anti- proliferation or pro-apoptotic functions. Airoldi *et al.* first demonstrated that IL-12Rβ2 could function as a tumor suppressor gene in human chronic B cell lymphoproliferative disorders, and IL-12 treatment in IL-12R transfected B lymphoma cells significantly inhibited cell proliferation and reduced tumorigenesis in animal model [15]. IL-12 induced and IL-12R mediated apoptosis has also been found in acute myeloid leukemia cells [16] and ovarian carcinoma cells [17]. IL-12 based tumor therapies have drawn greatly attention for the past 15 years and are apparently effective in prolonging the survival of cancer-bearing patients [18]. The situation seems to be the same for the other members of IL-12 family such as IL-23 and IL-27 [19].

We showed previously that spliced variants of IL-23R could generate defective IL-23R in various human tumor cell lines and different lung cancer tissues that might be a possible mechanism to account for the escape of immune surveillance in some human cancers [10]. IL-12Rβ2 can function as a tumor suppressor gene and can induce apoptosis in cancer cells. Due to the functional and structural similarity between IL-23R and IL-12Rβ2, we speculate that human IL-23R may also negatively regulate cell proliferation and promotes cell apoptosis. Indeed, in this study, we demonstrate that over-expression of human IL-23R could markedly induced cell apoptosis in both 293ET and HeLa cells. Mechanistic studies demonstrate that the classical intrinsic pathways might be activated in responding to *IL-23R* gene delivery. *IL-23R* gene might have the great potential to be developed as a therapeutic target against human cancers.

## 2. Results and Discussion

### 2.1. Overexpression of Human IL-23R Inhibits Cell Proliferation

**Figure 1.**
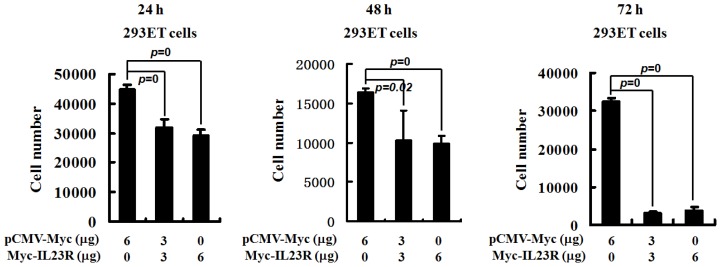
IL-23 receptor (IL-23R) inhibited the proliferation of transformed human embryonic kidney cell line 293ET cells. 293ET cells were transfected with increased doses (0, 3 and 6 μg) of IL-23R for 24, 48 and 72 h. The transfected cells were collected and assayed by CCK-8 kit. The reaction products were measured at 450 nm with a plate reader. The cell number for each dose was calculated using the established standard curve. The values represented as the mean ± SD from three to four independent experiments for each dose. Statistic analysis was performed with student’s *t* test. Statistical difference was considered to be significant if *p* < 0.05.

The IL-12R complex consists of two heterodimer chains named IL-12Rβ1 and IL-12Rβ2. The shared IL-12Rβ1 could also form heterodimer complex with IL-23R chain that is specifically recognized by IL-23. Since IL-12Rβ2 is a tumor suppressor gene, it might be true that IL-23R also possesses antiproliferative and proapoptotic effects. To test this assumption, CCK8 assay was performed to measure the cell growth ability affected by IL-23R. The 293ET cells transfected with different doses of IL-23R were collected at different time points. Figure 1 clearly demonstrates that the numbers of viable cells were significantly reduced as the doses of IL-23R increased after 24, 48 and 72 h post-transfections, indicating that the proliferation potentials of IL-23R transfected cells were markedly inhibited.

### 2.2. Over-Expression of Human IL-23R Induces Apoptosis in both 293ET and HeLa Cells

**Figure 2.**
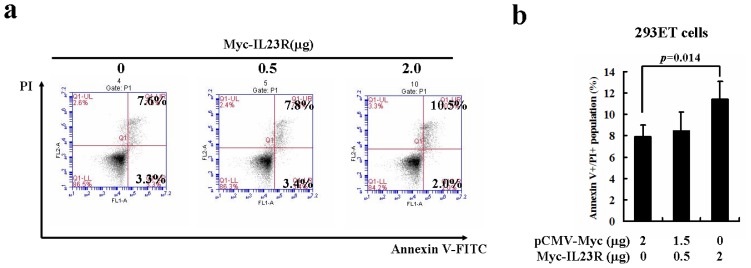
Human IL-23R induced cell apoptosis in human embryonic kidney cell line 293ET cells. (**a**) The detection of cell apoptosis in IL-23R transfected 293ET cells seeded on a 12 well culture plate was performed with Annexin V/Propidium iodide (PI) double staining followed by flow cytometric analysis; and (**b**) Quantitation of late apoptotic cells transfected by IL-23R. The transfected 293ET cells were stained and analyzed as described in A. Annexin V+/PI+ double positive cells were counted. Each value represented as mean ± SD from four independent transfections. Statistic analysis was performed with student’s *t* test. Statistical difference was considered significant if *p* < 0.05.

The next question that we tried to ask was whether IL-23R over-expression could affect cell survival. To this end, 293ET cells were transiently transfected with IL-23R. After 48-h posttransfection, the cells were collected and subjected to Annexin V and PI double staining. Subsequent flow cytometric analysis showed a marked increase in the population of Annexin V+/PI+ double positive cells in responding to higher dose IL-23R delivery (Figure 2a). Statistical analysis showed that higher dose delivery of IL-23R significantly increased the population of late apoptotic cells (Figure 2b). Since 293ET cell line is a cell line transformed by both SV40 large T antigen and EB virus nuclear antigen EBNA1, the biological properties of 293ET cells might not behave completely like normal cells. The non-cancer character of 293ET cell line could not exclude the possibility that normal cells/tissues are still not sensitized to IL-23R mediated apoptosis.

**Figure 3.**
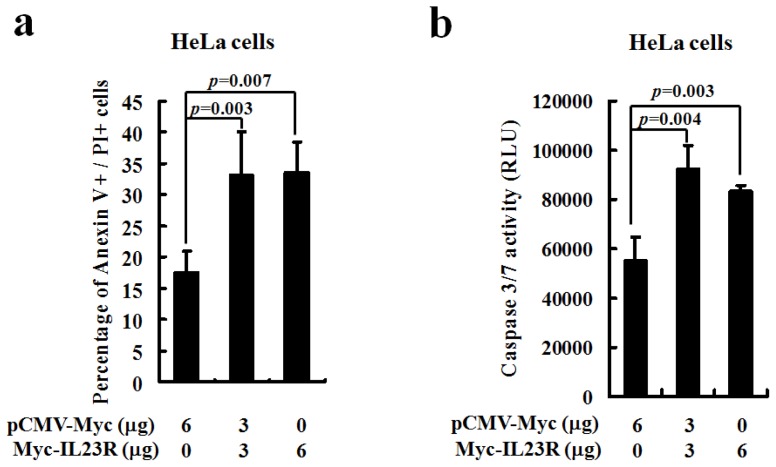
Human IL-23R induced cell apoptosis in human cervical cancer cell line HeLa cells. (**a**) Quantitation of late apoptotic cells. The detection of cell apoptosis in IL-23R transfected HeLa cells after 48 h transfection was performed with Annexin V/PI double staining followed by flow cytometric analysis. Annexin V+/PI+ double positive cells were counted. Each value represented as mean ± SD from four independent transfections. Statistic analysis was performed with student’s *t* test. Statistical difference was considered to be significant if *p* < 0.05; and (**b**) Detection of the apoptotic cells by measuring caspase 3/7 activities. After 48 h transfection, the transfected HeLa cells were dissolved into Caspase-Glo 3/7 Reagent. The reaction product was subjected to luminescence using a plate-reading luminometer. Each value is represented as mean ± SD from four independent transfections. Statistic analysis was performed with student’s *t* test. Statistical difference was considered to be significant if *p* < 0.05.

To further test its pro-apoptotic effect, the plasmid IL-23R DNAs were also transiently transfected into human cervical cancer cell line HeLa cells. Consistently, flow cytometric analysis shows that the Annexin V+/PI+ double positive HeLa cells were dramatically increased as the delivered dose of IL-23R increased (Figure 3a). In addition, the activities of caspase 3/7 effectors were also directly detected in transfected HeLa cells. The caspase 3/7 activities are monitored by aminoluciferin which is coupled with caspase 3/7 substrate cleavage site followed by luciferase detection. Indeed, a significant enhancement in caspase 3/7 activity was observed as the dose of transfected IL-23R increased (Figure 3b), indicating that IL-23R is able to activate the key effectors in the apoptotic pathway. Overall, our data indicates that IL-23R is indeed an apoptotic inducer that could activate the apoptotic processes in both 293ET and HeLa cells.

### 2.3. IL-23R Activates Cell Apoptosis through Intrinsic Pathway

**Figure 4.**
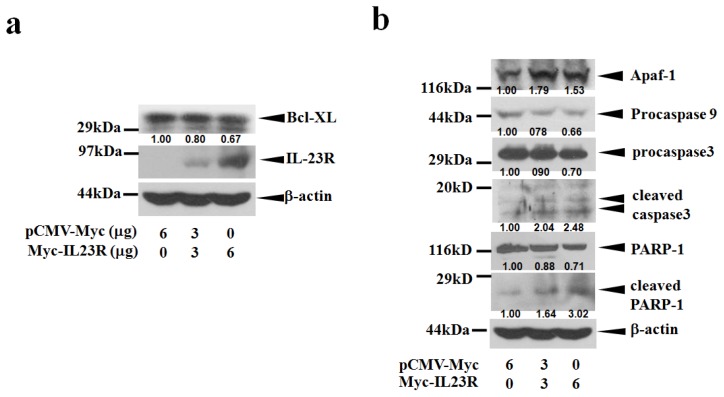
IL-23R mediated cell apoptosis in 293ET cells is associated with mitochrondia mediated intrinsic pathway. (**a**) IL-23R inhibited Bcl-xL expression in a dose dependent manner; and (**b**) The cell lysates prepared from IL-23R transfected 293ET cells after 48 h transfection were probed with anti-caspase 3, anti Apaf-1, anti caspase 9 and anti-PARP-1 antibodies and analyzed by Western blot analysis. Beta-actin gene expression was served as internal control. In both (**a**) and (**b**), the relative intensity of individual band was calculated based on the expression of β-actin by using the Quantity One program.

Since IL-23R was over-expressed in the targeted cells, IL-23R induced cell apoptosis might be associated with the activation of intrinsic mitochondria pathway. To test this hypothesis, cell lysates prepared from IL-23R transfected 293ET cells were first probed with anti-Bcl-xL antibody. Bcl-xL belongs to BCL2 family and exerts its anti-apoptotic function by preventing the oligomerization of BAX/BAK proteins at the mitochondrial outer membrane [20]. Indeed, over-expression of IL-23R in 293ET cells inhibited the expression levels of Bcl-xL proteins in a dose-dependent manner (Figure 4a). Further study indicated that increased delivery of IL-23R into 293ET cells also markedly promoted Apaf-1 expression and procaspase 9 cleavages that subsequently lead to the activation of the executioner caspase 3 (Figure 4b). In addition, a marked increase in the production of the cleaved PARP-1 was also detected as the doses of IL-23R increased. Thus, the data strongly indicates that IL-23R mediated apoptosis is associated with intrinsic mitochondrial pathway in 293ET cells.

**Figure 5.**
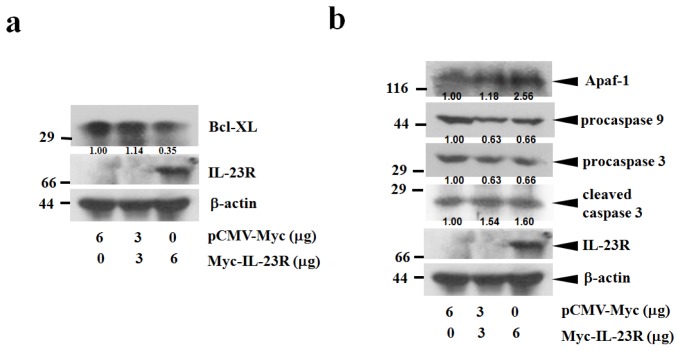
IL-23R mediated cell apoptosis in HeLa cells is also associated with mitochrondia mediated intrinsic pathway. (**a**) IL-23R inhibited Bcl-XL expression in a dose dependent manner; and (**b**) The cell lysates prepared from IL-23R transfected HeLa cells after 48 h transfection were probed with anti Apaf-1, anti caspase 9 and anti-caspase 3 antibodies and analyzed by Western blot analysis. β-actin gene expression was served as internal control. In both (**a**) and (**b**), the relative intensity of individual band was calculated based on the expression of β-actin by using the Quantity One program.

To further confirm the result, the same assay system was also applied to HeLa cells. In agreement with the results of 293ET cells, overexpression of IL23R significantly decreased *Bcl-xL* gene products in a dose dependent manner (Figure 5a). The dose dependent changes were also true for the increased Apaf-1 expression, and promoted caspase 9 and caspase 3 cleavages (Figure 5b). Therefore, the results strongly indicate that IL-23R mediated activation of intrinsic mitochondrial pathways might be a common mechanism responsible for the induction of cell apoptosis in mammalian cells.

### 2.4. IL-23R Inhibits both RAS/MAPK and STAT3 Signaling Pathways in both 293ET and HeLa Cells

**Figure 6.**
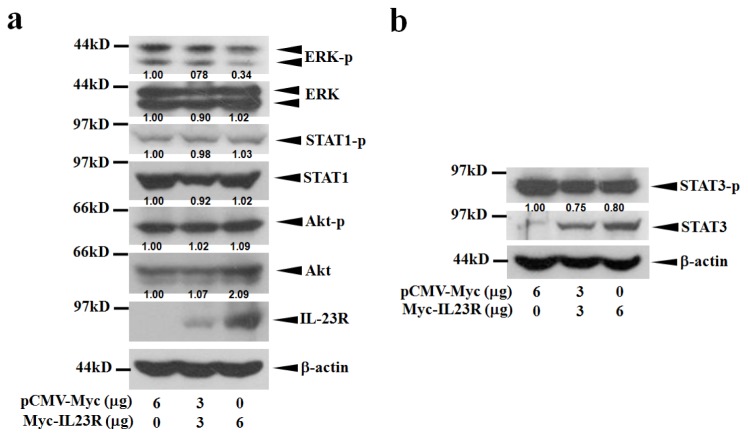
The mitogenic signaling pathways are repressed by IL-23R in 293ET cells. (**a**) Western blot analysis was performed using anti ERK, anti-phosphorylated ERK, anti STAT1, anti-phosphorylated STAT1, anti Akt, and anti-phosphoryled Akt antibodies. The tranfected Myc-IL-23R after 48 h transfection was visualized by anti Myc antibody. Beta-actin gene expression was served as internal control; and (**b**) Western blot analysis was performed using anti STAT3 and anti-phosphorylated STAT3 antibodies. β-actin gene expression was served as internal control. In both (**a**) and (**b**), the relative intensity of individual band was calculated based on the expression of β-actin by using the Quantity One program.

Cell apoptosis is frequently associated with the alterations of cell survival signaling pathways. To detect the influence of IL-23R mediated apoptosis on the activation of major mitogenic signaling pathways, western blot analysis was conducted to assay the phosphorylation levels of STAT1, STAT3, Akt and ERK in 293ET cells. Figure 6a shows that the activation of ERK was inhibited as the doses of IL-23R increased. Interestingly, the activations of STAT3 were inversely related to the protein levels of inactivated STAT3 which were upregulated with the increased doses of IL-23R (Figure 6b). However, no significant alterations were observed when the phosphorylation levels of both STAT1 and Akt were assayed (Figure 6a). The data indicates that IL-23R could markedly inhibit both RAS/MAPK and STAT3 signaling pathways but not STAT1 and Akt signaling pathways in 293ET cells.

**Figure 7.**
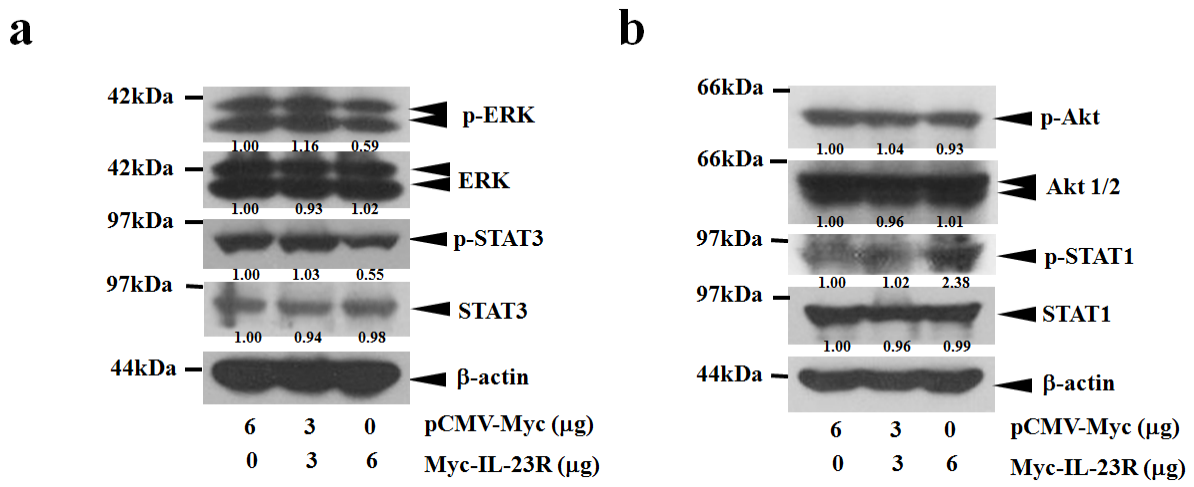
The intracellular signaling pathways are altered upon the overexpression of IL-23R in HeLa cells. (**a**) Western blot analysis was performed anti-phosphorylated extracellular regulated protein kinases (p-ERK), anti ERK, anti STAT3 and anti-phosphorylated STAT3 antibodies. β-actin gene expression was served as internal control; and (**b**) Western blot analysis was performed using using anti-phosphoryled Akt, anti Akt, anti-phosphorylated STAT1 and anti STAT1 antibodies. β-actin gene expression was served as internal control. In both (**a**) and (**b**), the relative intensity of the individual bands were calculated based on the expression of β-actin by using the Quantity One program.

To further elucidate the molecular mechanisms responsible for IL-23R mediated apoptosis, the cell survival signaling pathways were also assayed in transfected HeLa cells. Consistent with the previous results, the increased delivery of IL-23R markedly reduced the phosphorylation levels of both ERK and STAT3, demonstrating that IL-23R inhibited both ERK and STAT3 pathways in a dose-dependent manner (Figure 7a). However, no significant alteration in Akt phosphorylation could be detected as the dose of IL-23R increased (Figure 7b). Interestingly, increased delivery of IL-23R into HeLa cells did not reduced but markedly enhanced the phosphorylation level of STAT1, indicating that activation of STAT1 might be associated with cell apoptosis in HeLa cells (Figure 7b). Overall, the data strongly indicates that inhibition on both ERK and STAT3 signaling pathways might be the common mitogenic signaling pathways associated with IL-23R mediated apoptosis in mammalian cells.

### 2.5. The Effects of IL-23R Overexpression on Endogenous Expressions of IL-23 Complex and IL-12Rβ1 in both 293ET and HeLa Cells

**Figure 8.**
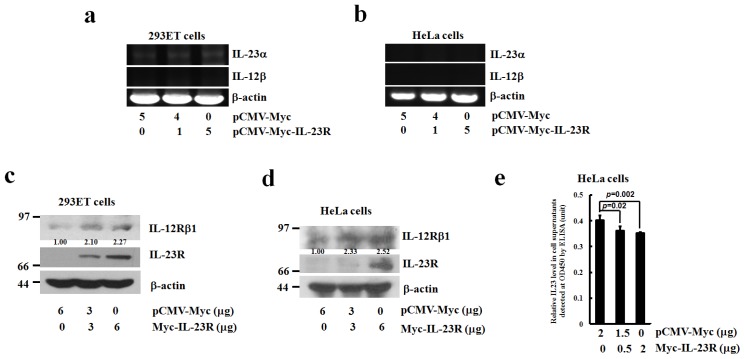
The expression levels of endogenous IL-23α, IL-12β and IL-12Rβ1 in responding to the overexpression of IL-23R. Semi-quantitative RT-PCR analysis was performed to detect the endogenous IL-23α and IL-12β expressions in both 293ET (**a**) and HeLa cells (**b**) in responding to exogenous delivery of IL-23R; the expression levels of endogenous IL-12Rβ1 were detected by western blot analysis on IL-23R transfected 293ET (**c**) and HeLa cells (**d**). The data is the representative of at least two independent transfection assays. The expression of β-actin was served as an internal control. The relative intensities of individual bands were calculated based on the expression of β-actin by using the Quantity One program; and (**e**) Detection of IL-23 production in HeLa cell supernatants in responding to IL-23R delivery by Enzyme Linked Immunosorbent Assay (ELISA). After 48 h transfection, the cell supernatants were collected from a 12 well plate and subjected to standard ELISA analysis. The reaction products were detected at 450 nm using a microtiter plate reader. Each value is represented as mean ± SD from four independent transfections. Statistic analysis was performed with student’s *t* test. Statistical difference was considered to be significant if *p* < 0.05.

To assay the endogenous expressions of IL-23 complex in responding to IL-23R overexpression, total RNAs were extracted from IL-23R transfected 293ET and HeLa cells. RT-PCR analysis revealed that no significant alterations in the expression of IL-23α subunit were observed as the delivered doses of IL-23R increased in 293ET cells, while no expression of IL-12β mRNA could be detected in the same condition (Figure 8a). Similarly, in IL-23R transfected HeLa cells, no significant enhancement of either IL-23α or IL-12β mRNAs could be detected (Figure 8b). In contrast, western blotting analysis demonstrates that increased delivery of IL-23R into either 293ET or HeLa cells significantly promoted IL-12Rβ1 expressions (Figure 8c,d). To further detect the driving force of IL-23R mediated apoptosis, cell supernatants were collected and assayed for the presence of IL-23 ligand by ELISA analysis. Figure 8e demonstrates an inverse correlation between the levels of extracellular IL-23 ligands versus the increased delivery of IL-23R inside the transfected cells, indicating that overexpression of IL-23R does not promote IL-23 production in HeLa cells. Collectively, the data indicates that overexpression of IL-23R might induce cell apoptosis in a ligand independent manner.

Some Bcl-2 family members, for instance Bcl-xL, could function as a negative regulator to counteract the cellular apoptotic process. Under certain circumstances, enhanced expressions of Bcl-xL promote tumor growth and subsequently lead to drug resistance that in turn affects the efficacy of chemotherapy. Therefore, Bcl-xL has long been considered as an ideal target for certain cancer treatments [20]. The expression level of Bcl-xL was regulated by many transcription factors such as Ets, NFκB, STAT3 and AP-1 [21], whereas ERK1/2 pathway could be the upstream regulator to be responsible for the activations of these transcriptional factors. Emerged evidence has strongly indicated that chemical mediated inhibition on ERK1/2 signaling pathway can greatly improve the efficacy of tumor chemotherapy by down-regulating the expression of Bcl-xL that often leads to the activation of intrinsic apoptotic pathway [22–26]. Therefore, it is likely that IL-23R could be developed as a new therapeutic target for certain cancer treatment.

## 3. Experimental Section

### 3.1. Cell Lines, Chemicals and Reagents

Human embryonic kidney cells expressing SV40T and EBNA1 (293ET) and human cervical cancer cell line (HeLa) were derived from the Cell Culture Center of Institute of Basic Medical Sciences, Chinese Academy of Medical Sciences (Beijing, China). Mouse anti-Myc, rabbit anti-caspase 3, rabbit anti-STAT3, rabbit anti-p-STAT3 goat anti-Akt1/2, rabbit anti-p-Akt1/2/3, rabbit anti-ERK1, rabbit anti-p-ERK and mouse anti β-actin were purchased from Santa Cruz Biotechnology (Santa Cruz, CA, USA). Rabbit anti-PARP1 was purchased from Epitomics Inc. (Burlingame, CA, USA). Rabbit anti-Apaf1 antibody was purchased from Beijing Biosynthesis Biotechnology (Beijing, China). Rabbit anti-STAT1 and Rabbit anti-p-STAT1 were purchased from Bioworld Technology Inc. (Bioworld, CA, USA). The peroxidase-conjugated secondary antibodies were from Zhongshan Biotechnology (Beijing, China). The full length of human IL-23R cDNA was isolated from the total RNAs of PBMC [10], and subcloned into the SalI/KpnI sites of pCMV-Myc vector to generate the eukaryotic expression vector pCMV-Myc-IL-23R whose sequence was subsequently confirmed by DNA sequencing analysis.

### 3.2. Cell Culture and Transfection

Cells were cultured in DMEM containing 10% fetal calf serum at 37 °C supplemented with 5% CO_2_. Transient transfection was performed using Vigofect (Vigorous Inc, Beijing, China) with the indicated plasmid DNAs. Cells seeded onto 6 well culture plates or 35 mm^2^ culture plates were received 0, 3 and 6 μg transfected pCMV-Myc-IL-23R DNAs, while cells plated onto 12 well culture plates were delivered with the doses of 0, 0.5 and 2 μg of pCMV-Myc-IL-23R DNAs. Then the transfected cells were continuously incubated for 48 h before harvesting.

### 3.3. Cell Proliferation Assay by Cell-Counting Kit-8 (CCK-8)

Cell proliferation was monitored by CCK-8 assay. Briefly, cells were cultured to a density of 5 × 10^4^ cells/mL and then certain amount cells were released and transferred into a 96-well plate after 24, 48 or 72 h post-transfection. Cell-counting kit-8 (Dojindo, Kumamoto, Japan) solution was added into each well. After 2.5 h incubation, the absorbance at 450 nm was measured using a microplate reader.

### 3.4. Flow Cytometric Analysis

Cell apoptosis was detected by Annexin V-FITC apoptosis detection kit (Beijing RuiBang XingYe Science & Technology Co., LTD, Beijing, China). After 48 h transfection, cells were released and washed with pre-cooled 1× PBS. Then the cell pellet was resuspended with 50 μL binding buffer followed by adding 5 μL Annexin V-FITC and 5 μL Propidium Iodide (PI). Then the cell suspension was incubated away from light for 15 min at room temperature. Another 150 μL binding buffer was added into the mixture before flow cytometric analysis by Accuri C6 (BD Biosciences, San Jose, CA, USA).

### 3.5. Caspase 3/7 Assay

Caspase-3/7 activity was detected using Caspase-Glo 3/7 assay kit (Promega, Madison, WI, USA). After 48 h transfection, cells were released and washed with pre-cooled 1× PBS. Then cells were transferred into a 96-well plate and the same amount of Caspase-Glo 3/7 Reagent was added to each well. The mixture was incubated at room temperature for 1 h. The luminescence of each sample was measure in a plate-reading luminometer.

### 3.6. Reverse Transcription-Polymerase Chain Reaction (RT-PCR)

Total RNAs were extracted from the cultured cells with TRIzol (Invitrogen, Carlsbad, CA, USA). The primers used in the RT-PCR reactions were listed in Table 1. One microgram of total RNAs was subjected to RT-PCR analysis using PrimeScript One Step RT-PCR Kit (Takara Biotechnology, Dalian, China). The reverse transcription was conducted at 42 °C for 35 min. After being denatured at 94 °C for 5 min, the reaction products were subsequently PCR amplified at 94 °C for 30 s, 55 °C for 30 s and 72 °C for 40 s for 25 cycles before extension at 72 °C for 10 min. The level of *β-actin* gene expression was served as internal control.

### 3.7. Western Blot Analysis

The transfected cells were lysed with gentle rotation in a lysis buffer (1% NP-40, 50 mM Tris-HCl (pH 7.5), 120 mM NaCl, 200 μM NaVO_4_, 1 μg/mL leupeptin, 1 μg/mL aprotinin, and 1 μM PMSF). Cell lysate for each sample was resolved on 12% SDS-PAGE followed by blotting onto Hybond nitrocelluar membrane (Amersham Biosciences, Freiburg, Germany). The transferred membrane was then probed with primary antibodies followed by relevant secondary antibodies conjugated to horseradish peroxidase. Detection was enhanced by chemiluminescence (Santa Cruz Biotechnology, Santa Cruz, CA, USA).

### 3.8. Enzyme Linked Immunosorbent Assay (ELISA)

The secretion of IL-23 ligand was monitored by human interleukin 23 (IL-23) ELISA Kit (Beijing Gersion Biotechnology Co. Ltd, Beijing, China). Briefly, after 48 h transfection, 10 μL of culture supernatant together with 40 μL of sample diluent were added into the testing samples that were well pre-coated with hIL-23 antibody. Then, 100 μL of HRP-conjugated reagent were added into each well and incubated at 37 °C for 60 min. After aspirating and washing, 50 μL of chromogen A and B were added into each well and incubated away from light at 37 °C for 15 min. Finally, about 50 μL of stopping solution was added to each reaction before detection at 450 nm using a microtiter plate reader.

### 3.9. Statistical Analysis

All values were calculated as mean ± standard deviation (SD) from four independent experiments. The statistical difference between the assayed group and the standard group was subject to student’s *t* test (two-tailed, unpaired). The calculated difference was considered significant as the *p* value < 0.05 or < 0.01.

## 4. Conclusions

In the current study, we found that over-expression of IL-23R not only inhibited the proliferation of 293ET and HeLa cells, but also effectively induced apoptosis in both 293ET and HeLa cells. Over-expression of IL-23R markedly down-regulated the expression of anti apoptotic factor Bcl-xL and activated both the initiator and executioner of the intrinsic apoptotic pathway in both cell lines. This process is likely independent of IL-23 ligand stimulation. Our data reveals that RAS/MAPK signaling pathway that is connected with STAT3 signaling pathway might be the central mitogenic pathway negatively regulated by IL-23R in transformed or cancer derived mammalian cells. Our results highlight a previously unrecognized role of IL-23R that can be potentially explored as a therapeutic agent against human cancers.

## Figures and Tables

**Table 1.** Primers used in RT-PCR analysis.

Gene Name	GenBank ID	Forward Primer (5′→3′)	Reverse Primer (5′→3′)	Size of Product (bp)
*β-actin*	BC009275	cacactgtgcccatctacga	ctgcttgctgatccacatct	600
*IL-23a*	NM_016584	aagtggaagtgggcagagat	atccttgagctgctgccttt	664
*IL-12β*	NM_002187	ttctggacgtttcacctgct	tttttgcggcagatgaccgt	500

## References

[1] Jones L.L., Vignali D.A. (2011). Molecular interactions within the IL-6/IL-12 cytokine/receptor superfamily. Immunol. Res.

[2] van Wanrooij R.L., Zwiers A., Kraal G., Bouma G. (2012). Genetic variations in interleukin-12 related genes in immune-mediated diseases. J. Autoimmun.

[3] Vignali D.A., Kuchroo V.K. (2012). IL-12 family cytokines: Immunological playmakers. Nat. Immunol.

[4] Chua A.O., Chizzonite R., Desai B.B., Truitt T.P., Nunes P., Minetti L.J., Warrier R.R., Presky D.H., Levine J.F., Gately M.K. (1994). Expression cloning of a human IL-12 receptor component. A new member of the cytokine receptor superfamily with strong homology to gp130. J. Immunol.

[5] Presky D.H., Yang H., Minetti L.J., Chua A.O., Nabavi N., Wu C.Y., Gately M.K., Gubler U. (1996). A functional interleukin 12 receptor complex is composed of two β-type cytokine receptor subunits. Proc. Natl. Acad. Sci. USA.

[6] Oppmann B., Lesley R., Blom B., Timans J.C., Xu Y., Hunte B., Vega F., Yu N., Wang J., Singh K. (2000). Novel p19 prtein engages IL-12p40 to form a cytokine, IL-23, with biological activities similar as well as distinct from IL-12. Immunity.

[7] Parham C., Chirica M., Timans J., Vaisberg E., Travis M., Cheung J., Pflanz S., Zhang R., Singh K.P., Vega F. (2002). A receptor for the heterodimeric cytokine IL-23 is composed of IL-12Rbeta1 and a novel cytokine receptor subunit, IL-23R. J. Immunol.

[8] Pflanz S., Hibbert L., Mattson J., Rosales R., Vaisberg E., Bazan J.F., Phillips J.H., McClanahan T.K., de Waal Malefyt R., Kastelein R.A. (2004). WSX-1 and glycoprotein 130 constitute a signal-transducing receptor for IL-27. J. Immunol.

[9] Collison L.W., Delgoffe G.M., Guy C.S., Vignali K.M., Chaturvedi V., Fairweather D., Satoskar A.R., Garcia K.C., Hunter C.A., Drake C.G. (2012). The composition and signaling of the IL-35 receptor are unconventional. Nat. Immunol.

[10] Zhang X.Y., Zhang H.J., Zhang Y., Fu Y.J., He J., Zhu L.P., Wang S.H., Liu L. (2006). Identification and expression analysis of alternatively spliced isoforms of human interleukin-23 receptor gene in normal lymphoid cells and selected tumor cells. Immunogenetics.

[11] Li J., Zhang L., Zhang J., Wei Y., Li K., Huang L., Zhang S., Gao B., Wang X., Lin P. (2013). Interleukin 23 regulates proliferation of lung cancer cells in a concentration-dependent way in association with the interleukin-23 receptor. Carcinogenesis.

[12] Tao Y.P., Wang W.L., Li S.Y., Zhang J., Shi Q.Z., Zhao F., Zhao B.S. (2012). Associations between polymorphisms in *IL-12A*, *IL-12B*, *IL-12Rβ1*, *IL-27* gene and serum levels of IL-12p40, IL-27p28 with esophageal cancer. J. Cancer Res. Clin. Oncol.

[13] Ferretti E., Montagna D., Di Carlo E., Cocco C., Ribatti D., Ognio E., Sorrentino C., Lisini D., Bertaina A., Locatelli F. (2012). Absence of IL-12Rβ2 in CD33(+)CD38(+) pediatric acute myeloid leukemia cells favours progression in NOD/SCID/IL2RgammaC-deficient mice. Leukemia.

[14] Cardenes M., Angel-Moreno A., Fieschi C., Sologuren I., Colino E., Molines A., Garcia-Laorden M.I., Campos-Herrero M.I., Andujar-Sanchez M., Casanova J.L. (2010). Oesophageal squamous cell carcinoma in a young adult with IL-12R beta 1 deficiency. J. Med. Genet.

[15] Airoldi I., di Carlo E., Banelli B., Moserle L., Cocco C., Pezzolo A., Sorrentino C., Rossi E., Romani M., Amadori A. (2004). The IL-12Rβ2 gene functions as a tumor suppressor in human B cell malignancies. J. Clin. Invest.

[16] Ferretti E., di Carlo E., Cocco C., Ribatti D., Sorrentino C., Ognio E., Montagna D., Pistoia V., Airoldi I. (2010). Direct inhibition of human acute myeloid leukemia cell growth by IL-12. Immunol. Lett.

[17] Gorelik E., Edwards R.P., Feng X., Marrangoni A.M., Grandis J.R., Drenning S.D., Velikokhatnaya L., Kwon J.A., Lokshin A.E. (2004). IL-12 receptor-mediated upregulation of FasL in human ovarian carcinoma cells. Int. J. Cancer.

[18] Bubenik J. (2011). Interleukin 12 in cancer treatment. Folia. Biol. (Praha).

[19] Engel M.A., Neurath M.F. (2011). Anticancer properties of the IL-12 family—Focus on colorectal cancer. Curr. Med. Chem.

[20] Kang M.H., Reynolds C.P. (2009). Bcl-2 inhibitors: Targeting mitochondrial apoptotic pathways in cancer therapy. Clin. Cancer Res.

[21] Sevilla L., Zaldumbide A., Pognonec P., Boulukos K.E. (2001). Transcriptional regulation of the *bcl-x* gene encoding the anti-apoptotic Bcl-xL protein by Ets, Rel/NFkappaB, STAT and AP1 transcription factor families. Histol. Histopathol.

[22] Choi H.J., Smithgall T.E. (2004). HIV-1 Nef promotes survival of TF-1 macrophages by inducing Bcl-XL expression in an extracellular signal-regulated kinase-dependent manner. J. Biol. Chem.

[23] Jazirehi A.R., Vega M.I., Chatterjee D., Goodglick L., Bonavida B. (2004). Inhibition of the Raf-MEK1/2-ERK1/2 signaling pathway, Bcl-xL down-regulation, and chemosensitization of non-Hodgkin’s lymphoma B cells by Rituximab. Cancer Res.

[24] Peng C.L., Guo W., Ji T., Ren T., Yang Y., Li D.S., Qu H.Y., Li X., Tang S., Yan T.Q. (2009). Sorafenib induces growth inhibition and apoptosis in human synovial sarcoma cells via inhibiting the RAF/MEK/ERK signaling pathway. Cancer Biol. Ther.

[25] Lu X., Tang X., Guo W., Ren T., Zhao H. (2010). Sorafenib induces growth inhibition and apoptosis of human chondrosarcoma cells by blocking the RAF/ERK/MEK pathway. J. Surg. Oncol.

[26] Nishioka C., Ikezoe T., Yang J., Yokoyama A. (2009). Inhibition of MEK signaling enhances the ability of cytarabine to induce growth arrest and apoptosis of acute myelogenous leukemia cells. Apoptosis.

